# SPECT imaging of glioma with radioiodinated CLINDE: evidence from a mouse GL26 glioma model

**DOI:** 10.1186/s13550-015-0092-4

**Published:** 2015-03-13

**Authors:** Stergios Tsartsalis, Noé Dumas, Benjamin B Tournier, Tien Pham, Marcelle Moulin-Sallanon, Marie-Claude Grégoire, Yves Charnay, Philippe Millet

**Affiliations:** Vulnerability Biomarkers Unit, Division of General Psychiatry, Department of Mental Health and Psychiatry, University Hospitals of Geneva, Chemin du Petit-Bel-Air 2, CH1225 Geneva, Chêne-Bourg Switzerland; Department of Psychiatry, University of Geneva, 1 rue Michel-Servet, CH1211 Geneva 4, Switzerland; ANSTO LifeSciences, Australian Nuclear Science and Technology Organisation, New Illawarra Road, Sydney, NSW 2234 Australia; INSERM, J. Fourier University, INSERM Unit 1039, Domaine de la Merci, 38700 La Tronche, France

**Keywords:** TSPO, CLINDE, Glioma, SPECT

## Abstract

**Background:**

Recent research has demonstrated the potential of 18-kDa translocator protein (TSPO) to serve as a target for nuclear imaging of gliomas. The aim of this study was to evaluate SPECT imaging of GL26 mouse glioma using radioiodinated CLINDE, a TSPO-specific tracer.

**Methods:**

GL26 cells, previously transfected with an enhanced green fluorescent protein (EGFP)-expressing lentivirus, were stereotactically implanted in the striatum of C57/Bl6 mice. At 4 weeks post-injection, dynamic SPECT scans with [^123^I]CLINDE were performed. A displacement study assessed specificity of tracer binding. SPECT images were compared to results of autoradiography, fluorescence microscopy, *in situ* nucleic acid hybridization, histology, and immunohistochemistry. Western blotting was performed to verify TSPO production by the tumor.

**Results:**

Specific uptake of tracer by the tumor is observed with a high signal-to-noise ratio. Tracer uptake by the tumor is indeed 3.26 ± 0.32 times higher than that of the contralateral striatum, and 78% of the activity is displaceable by unlabeled CLINDE. Finally, TSPO is abundantly expressed by the GL26 cells.

**Conclusions:**

The present study demonstrates the feasibility of [^123^I]CLINDE SPECT in translational studies and underlines its potential for clinical glioma SPECT imaging.

## Background

The 18-kDa translocator protein (TSPO) is primarily located at the contact sites between the inner and outer mitochondrial membranes, as a component of the mitochondrial permeability transition pore. Although its expression in healthy brain is minimal, it increases substantially in neuroinflammatory conditions [1] as well as in brain malignancies [2-4]. In the case of brain tumors, TSPO is predominantly expressed by neoplastic cells, thus, it is regarded as a potential central nervous system (CNS) cancer imaging biomarker.

Radioiodinated CLINDE is a highly-specific SPECT TSPO tracer that has been already evaluated in animal models of neuroinflammation [5,6]. The aim of this study was to investigate the potential use of radioiodinated CLINDE as a brain tumor SPECT imaging tracer in the mouse GL26 glioma model using *in vivo* high-resolution SPECT [7]. To validate our approach, we 1) compared i*n vivo* measurements to *ex vivo* autoradiography and histology; 2) evaluated the specificity of tracer binding (*in vivo* displacement procedure); and 3) confirmed the presence of TSPO mRNA by means of *in situ* hybridization, as well as TSPO protein levels produced by the tumor by means of immunohistochemistry and western blotting, respectively.

## Methods

All chemicals were purchased from Sigma-Aldrich (St. Gallen, Switzerland), unless otherwise specified. CLINDE precursor was provided by the Australian Nuclear Science and Technology Organization (ANSTO).

GL26 mouse glioma cells were kindly provided by Prof. L. Zitvogel (Institut G. Roussy, Paris, France). They were routinely cultured in DMEM, supplemented with 10% fetal bovine serum (FBS) and 1 mM sodium pyruvate (Life technologies, Zug, Switzerland). A stable GL26 cell line expressing enhanced green fluorescent protein (EGFP) was obtained by a lentiviral vector transfection according to the manufacturer instructions (ViraPower Lentiviral Expression System, Life technologies, Zug, Switzerland). Blasticidin (from 2 to 5 μg/ml of culture medium during 2 weeks) was used for the selection of clone of GL26 cells expressing EGFP. GL26-EGFP cells (50.000 cells in 2.5 μl of culture medium/injection) were stereotactically implanted in the striatum of adult male C57/Bl6 mice (Janvier Laboratories, Saint Berthevin, France, *n* = 10), weighing 28.2 ± 3.2 g, under isoflurane (4% induction and 1.5% to 2% maintenance) anesthesia. Three of these animals received a concurrent second injection of culture medium only in the contralateral striatum. All animal experiments were approved by the Animal Ethics Committee of the Canton of Geneva and were in accordance with the European Union regulations on animal research.

[^123^I]CLINDE were labeled as previously described [8]. At 4 weeks post-injection, mice were injected with 62.24 ± 18.90 MBq of radiotracer and underwent a dynamic SPECT session with the U-SPECT-II scanner (MILABS, Utrecht, Netherlands), using a list mode acquisition protocol consisting of 60 frames of 84 s. For the displacement study, four out of ten mice were injected with unlabeled CLINDE (10 mg/kg) at the 82th minute post-injection of the labeled tracer. Total scan duration was 165 min.

SPECT tomograms were reconstructed with an ordered subsets expectation maximization (OSEM) algorithm (using four subsets and six iterations). SPECT data were corrected for radioactive decay, but no correction of attenuation and scatter was applied. A 0.4-mm FWHM filter was applied on images. The dynamic SPECT images were first averaged over time frames between 50 and 80 min in order to enhance visualization of the different structures. These images were manually co-registered to a mouse magnetic resonance imaging (MRI) and volume of interest (VOI) template [9] that was implemented in PMOD software (version 3.6, 2014, PMOD Technologies Ltd, Zurich, Switzerland), and the co-registration parameters were employed for dynamic image co-registration. Standardized uptake values (SUV) of tumor and contralateral striatum radioactivity were extracted from averaged frames between 50 and 80 min, using PMOD and compared by means of *t*-test (Microsoft Excel, 14.4.7).

Mice were sacrificed after the end of the scan session at 4 weeks post-implantation of GL26 cells. Brains were harvested and frozen in isopentane (−20°C) and serial coronal sections were taken. Brain sections were used for *ex vivo* autoradiography, fluorescence microscopy, Nissl staining, immunohistochemistry, and *in situ* hybridization. Two digoxigenin-labeled riboprobes complementary to the Mus musculus translocator protein (TSPO) mRNA sequences (NM-00977.4: coding sequences 102-380 and 322-577, respectively) were used. The cDNA amplicons from total RNA extracted from a mouse brain cortex were inserted in a TOPO-pCR4 vector (Invitrogen, Carlsbad, CA, USA) followed by linearization and *in vitro* transcription in presence of dUTP-digoxigenin (Roche, Basel, Switzerland) according to the manufacturer instructions (Invitrogen, Carlsbad, CA, USA). The *in situ* hybridization histochemistry procedures were previously reported in detail [10]. The use of sense probes, in serial tissue sections, resulted in the absence of any hybridizing signal whereas the two antisense independent probes mentioned above gave the same patterns of labeling. Thus, these data were in favor of the specificity of the hybridizing signal for TSPO mRNA visualized in the tumor tissue sections. TSPO-immunoreactivity expressed by glioma cells was further suggested by immunohistochemistry and the TSPO-immunoreactive band seen in a Western blot using an anti-TSPO antibody (goat anti-mouse TSPO antibody LS-B5755, LifeSpan Biosciences, Inc., Seattle, WA, USA; working dilution 1:1,000). The Western blotting procedure was published with detail elsewhere [11].

## Results

**Figure 1:**
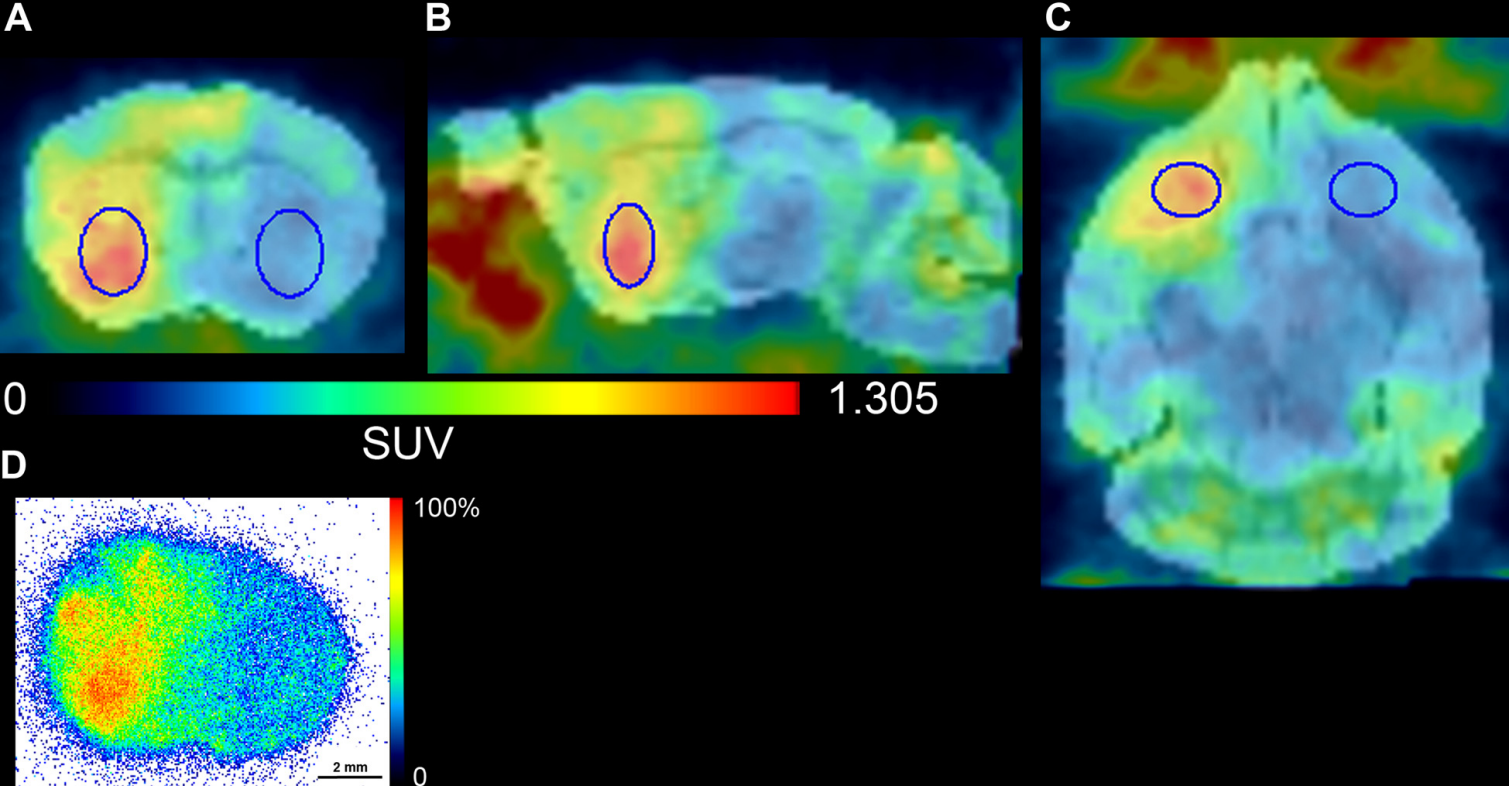
***In vivo*** **SPECT imaging, histology and** ***ex vivo*** **autoradiography.** *In vivo* SPECT image (summed frames between 50 and 80 min of scan), co-registered with a mouse brain MRI template, obtained from one mouse bearing the GL26 tumor in the coronal **(A)**, sagittal **(B)**, and axial **(C)** planes. VOIs corresponding to tumor and contralateral brain tissue are also depicted. **(D)** *Ex vivo* autoradiography of a corresponding brain section from the same mouse. Color scale refers to percentage of maximal activity in the image.

**Figure 2:**
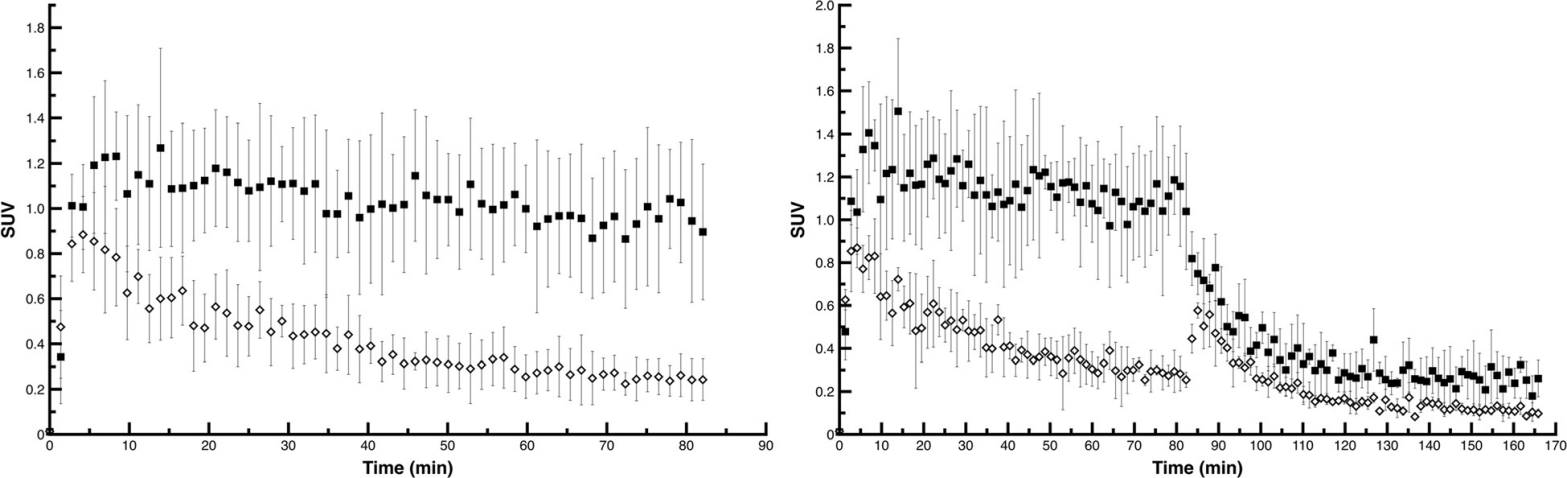
**Time-activity curves and displacement experiments.** TACs, corresponding to an oval-shaped VOI defined manually on the tumor of this mouse and an equal volume VOI placed on the contralateral hemisphere (left part, data represent mean ± SD of results from seven mice). Displacement with a saturating concentration of unlabeled CLINDE (10 mg/kg) was performed at 82 min of scan demonstrating that about 78% of the activity is displaceable, suggesting a significant proportion of specific binding (right part, data represent mean ± SD of results from four mice).

Figure 1A,B,C shows an *in vivo* SPECT image (averaged over frames corresponding to 50 to 80 min post-injection of tracer), co-registered to the mouse MRI template. A corresponding coronal brain section after *ex vivo* autoradiography is presented in Figure 1D. Figure 2 (left part) illustrates the tissue-activity curves (TACs) extracted from the dynamic images of seven mice using two circular VOIs manually delineated on the tumor as well as an equal size VOI on the contralateral striatum. A rapid tracer uptake is observed in the initial frames post-injection followed by washout that, for the contralateral VOI, is almost complete and rapid. Analysis of averaged image frames between 50 and 80 min post-injection across different experimental subjects demonstrated that the tumor side (mean SUV 1.04 ± 0.22) presents a level of radioactivity 3.26 ± 0.32 times higher than that of the contralateral side (mean SUV 0.32 ± 0.08, *p* < 0.001). TACs of the dynamic scan presented in Figure 2 (right part) also depict the result of a displacement experiment in which 10 mg/kg of unlabeled compound were injected at 82 min post-injection. Radioactivity kinetic pattern in the tumor VOI reveals that about 78% of the radioactivity is displaceable.

**Figure 3:**
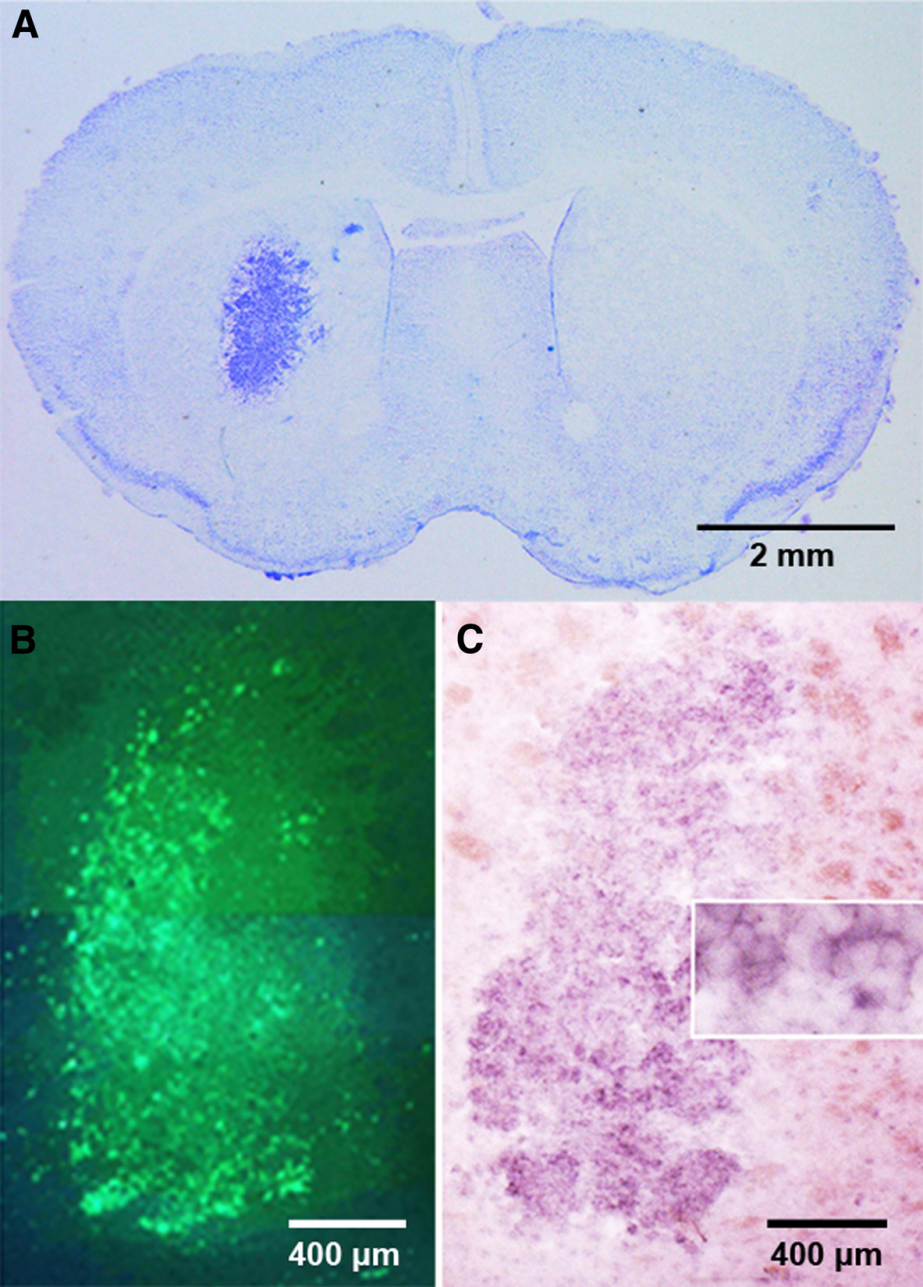
**Fluorescence microscopy and** ***in situ*** **mRNA hybridization. (A)** Coronal brain section of GL-26 tumor-bearing mouse visualized after Nissl staining. **(B)** A coronal brain section of another mouse visualized under fluorescence microscopy depicting the EGFP-expressing GL26 cells. **(C)** Result of *in situ* TSPO mRNA hybridization performed on an adjacent brain section (box depicts part of the slice in a greater magnification).

Figure 3A presents a coronal section of a tumor-bearing mouse after Nissl staining while Figure 3B depicts brain sections from another mouse that was analyzed by means of fluorescence microscopy and (Figure 3C) *in situ* hybridization with specific probes that demonstrate the expression of the TSPO mRNA by the tumor.

**Figure 4:**
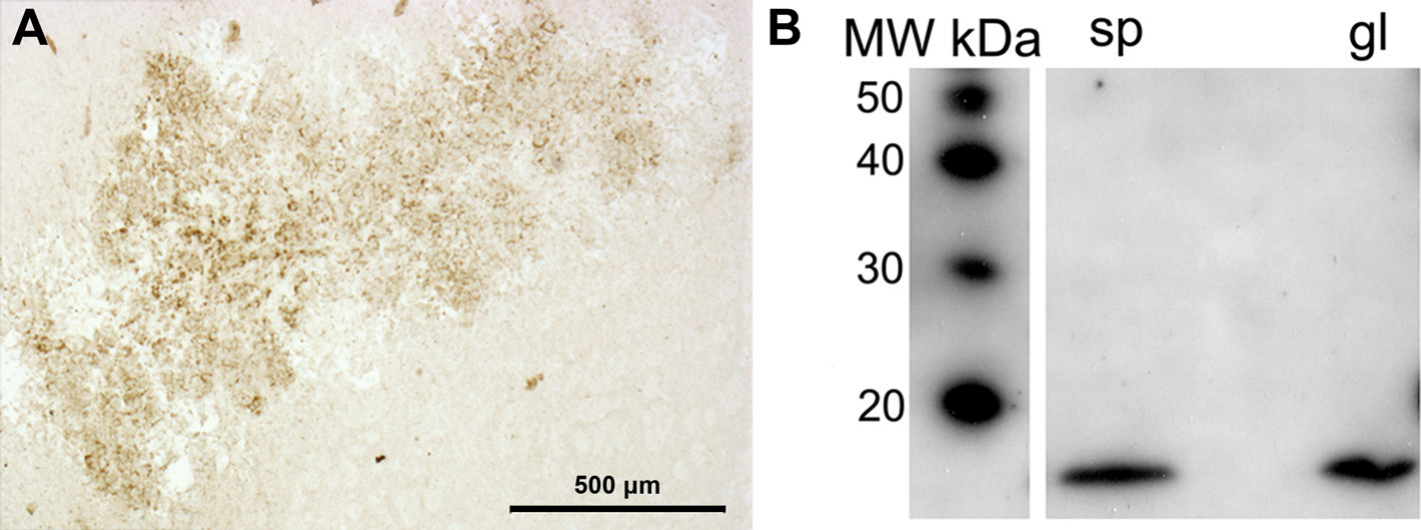
**Immunohistochemistry and Western blotting. (A)** Immunohistochemical analysis of a brain section from a mouse showing TSPO expression by tumor cells. **(B)** Western blotting assay demonstrates TSPO expression from GL26 tumor tissue (gl) as compared to spleen tissue (sp), used as a positive control. Molecular weight of the bands corresponds to 18 kDa, as demonstrated with the use of a molecular weight probe (left-most column).

Finally, immunohistochemical analysis (Figure 4A) shows TSPO-immunoreactivity in most of the tumor cells. Western blotting (Figure 4B) analyses of tumor tissue extracts compared to spleen extracts (as positive control) show a similar 18-kDa band confirming the presence of TSPO protein in these cell populations.

## Discussion

Standard clinical practice in brain tumor imaging includes MRI that is considered the ‘gold standard’ presenting high spatial resolution but failing to provide sufficient information on the histopathological and molecular profile of the malignant lesions [12]. On the contrary, nuclear imaging approaches such as positron emission tomography (PET) and SPECT may provide information on functional features of the disease such as metabolic activity and the expression of molecules specifically associated with this type of pathological processes of the CNS, one of them being TSPO. There is now evidence that TSPO expression might serve as a biomarker of tumor progression, metastatic potential, and overall prognosis in several human tumors [13].

TSPO imaging in glioma has already been assessed using PET through a series of studies using different [^11^C]- or [^18^ F]-labeled tracers [2-4,14]. Buck et al. [14] showed specific [^18^ F]PBR06 uptake that closely correlated with TSPO expression in a rat glioma model. Similarly, [^18^ F]DPA-714 preclinical imaging in rat models gave encouraging results [2,3]. Winkeler et al. [2] showed that neoplastic cells produce the majority of TSPO expressed by the tumor. In this study, TSPO expression by GL26 cells, as assessed by means of *in situ* hybridization for TSPO mRNA and fluorescence microscopy, supports the aforementioned observation.

Our study highlights the potential of SPECT imaging in clinical neuro-oncology. In fact, SPECT is frequently employed in clinical neuroscience: in the domain of neuro-oncology, recent meta-analyses of clinical studies demonstrate that SPECT, due to its high specificity, is perhaps the best imaging modality to differentiate between cerebral regions due to tumor recurrence or radiotherapy-induced necrosis [15,16]. Regarding TSPO imaging in particular, Awde et al. proposed that TSPO imaging could serve as a molecular biomarker of glioma progression and even response to treatment [17]. However, regarding human glioma imaging using TSPO-specific radiotracers, one has to take into account that not all types of glioma show equally high levels of TSPO expression as glioblastoma [4,18] and this element could be exploited for the differential diagnosis of different types of these tumors. In addition, fundamental differences between SPECT and PET render the former much more readily available in clinical settings due to the long half-lives of radionuclides employed in SPECT (e.g., 13.2 h for ^123^I compared to 110 min for ^18^F and 20 min for ^11^C, two widely employed PET radionuclides) and its lower cost. Similarly, in the experimental level, the emergence of new preclinical SPECT scanners provides sub-millimeter spatial resolution, superior to that of small animal PET and has rendered SPECT a particularly powerful tool for small animal research [19]. This, along with the aforementioned advantages of SPECT in the practical level (availability of radionuclides, lower cost), propose that employing radioiodinated CLINDE could be considered an attractive alternative to PET TSPO tracers already described above, in experimental and - potentially - in clinical settings. Indeed, in the present study, we demonstrate a specific binding of radioiodinated CLINDE to GL26 glioma, where TSPO expression is abundant. Tracer uptake by the tumor is 3.26 times higher than in the contralateral hemisphere, indicating a good signal-to-noise ratio. *In vivo* SPECT images, *ex vivo* autoradiography, and fluorescence microscopy show comparable results regarding tumor anatomy. A displacement study confirmed the specificity of tracer binding on TSPO as about 78% of the activity in the tumor was displaceable by the unlabeled compound. In Figure 2 (right part), an augmentation of the activity in the striatum that is contralateral to the one with the tumor after displacement with CLINDE is observed. It could be attributed to a displacement of the radiotracer from extracerebral tissues where TSPO expression is abundant [1] and entry into the brain. Finally, tracer uptake from tumor is not attributable to the lesion produced by the stereotactic injection of GL26 cells, as contralateral striatum, injected with culture medium alone, did not reveal but a negligible uptake (data not shown).

## Conclusions

To our knowledge, this is the first study on a potential TSPO SPECT glioma tracer. We believe that, given its specificity and its signal-to-noise ratio, our results demonstrate the quality of [^123^I]CLINDE SPECT imaging of glioma in translational studies and are indicative of its potential value in a clinical setting.
